# Lignans from *Machilus thunbergii* as Thymic Stromal Lymphopoietin Inhibitors

**DOI:** 10.3390/molecules26164804

**Published:** 2021-08-08

**Authors:** Hyeji Shin, Yoo Kyong Han, Youngjoo Byun, Young Ho Jeon, Ki Yong Lee

**Affiliations:** College of Pharmacy, Korea University, Sejong 30019, Korea; hjshin90@korea.ac.kr (H.S.); kkoo_@naver.com (Y.K.H.); yjbyun1@korea.ac.kr (Y.B.); yhjeon@korea.ac.kr (Y.H.J.)

**Keywords:** *Machilus thunbergii*, TSLP, STAT5, allergic disease, lignan

## Abstract

Thymic stromal lymphopoietin (TSLP) plays an important role in the pathophysiology of various allergic diseases that are mediated by T helper cell type-2 (Th2) responses, including asthma and atopic dermatitis. The primary focus of this study was the identification of potent inhibitors of the TSLP signaling pathway for potential therapeutic use. The 80% methanol extract of *Machilus thunbergii* bark significantly inhibited the signal transducer and activator of transcription 5 (STAT5) phosphorylation in human mast cell (HMC)-1 cells. Through activity-guided isolation, three lignans (**1**–**3**) were obtained and identified as (+)-galbelgin (**1**), *meso*-dihydroguaiaretic acid (**2**), and machilin A (**3**). Among them, two lignans (**1** and **2**) significantly inhibited STAT5 phosphorylation and TSLP/TSLPR interaction, as determined by ELISA. Our results indicated that lignans isolated from *M. thunbergii* are a promising resource for the treatment of allergic diseases.

## 1. Introduction

The prevalence of allergic diseases, such as asthma, rhinitis, and atopic dermatitis, is rapidly increasing due to continuous antigen exposure caused by changes in the environment, industrialization, and pollution. Thymic stromal lymphopoietin (TSLP), an epithelial cell-derived cytokine, plays a key role in allergic diseases promoted by dendritic cell-mediated T helper cell type-2 (Th2) [1,2,3]. When exposed to allergens, epithelial cells stimulate the production of cytokines, such as TSLP, interleukin-25 (IL-25), and IL-33. TSLP binds to the heterodimeric receptor complex of TSLP receptor (TSLPR) subunit and interleukin-7 receptor α chain (IL-7Rα). The binding induces the phosphorylation and activation of Janus kinases (JAKs) and the signal transducers and activators of transcription 5 (STAT5), leading to an allergic response as a result of the overproduction of Th2 cells [1,4,5]. Hence, TSLP signaling inhibitors can be used as materials for the development of allergy drugs. Common allergy drugs (antihistamines, steroids, and immunosuppressants) are useful only for symptomatic treatment and have side effects that include vomiting, headache, and dizziness when taken over a long period of time [6,7]. In addition, allergic diseases may continuously develop and reappear throughout life, with frequent recurrences and exacerbations affecting the quality of life [7,8]. Therefore, there is a need to develop a safe treatment that can be used for long periods of time, as well as being a fundamental treatment. Natural products capable of synergic effects due to the diversity of chemical structures have been suggested as alternatives to allergy drugs [9].

*Machilus thunbergii* (*M. thunbergii*) Sieb. et Zucc. (Lauraceae) is an evergreen broad-leaved tree up to 20 m tall, and its bark is dark brown with gray markings. It grows mainly in broad-leaved forests below 800 m and is distributed in warm regions such as Korea, China, Japan, and Taiwan [10,11]. The bark of *M. thunbergia* has been used in folk medicine for the treatment of abdominal pain and distension, leg edema, and anti-inflammatory responses [10,12]. Its bark consists of lignans (machilin A–I, licarlin A and B, and *meso*-dihydroguaiaretic acid), flavonoids (kaempferol, quercetin, and taxifolin), and butanolides (obtusilactone B, linderanolide, litsenolide A2, B1, and B2) [13,14,15,16,17], which have been reported to exert hepatoprotective [18], neuroprotective [19,20], antibacterial [21], and anticancer activities [22].

Seo et al. investigated the antiallergic effect of *M. thunbergii* extracts in vitro and found that the extracts of the whole plant, leaf, root, stem, fruit, and flower of *M. thunbergii* significantly inhibit the secretion of immunoglobulin (Ig) E in U266 cells [23]. In a previous study, an 80% methanol extract of the bark of *M. thunbergii* Sieb. et Zucc. significantly inhibited STAT5 phosphorylation in human mast cell (HMC)-1 cells. However, to the best of our knowledge, researchers have yet to investigate whether a direct correlation exists between the chemical composition and antiallergic activity of *M. thunbergii*. Therefore, the main objective of this study was to isolate and analyze antiallergic active constituents from the bark of *M. thunbergii*.

## 2. Results and Discussion

**Figure 1:**
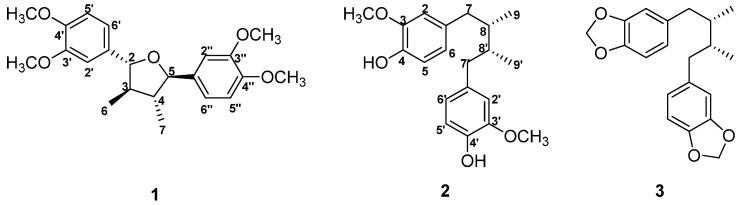
Chemical structures of isolated compounds **1**–**3** of *Machilus thunbergii* (*M. thunbergii*).

In a study to identify antiallergic active constituents from natural products, 80% MeOH extracts of the bark of *M. thunbergii* exhibited significant TSLP inhibitory activity in the pSTAT5 assay (69.2% at 100 µg/mL; Table 1). In an attempt to evaluate active constituents obtained from the bark of *M. thunbergii*, three lignans were isolated and identified as (+)-galbelgin (**1**) [24,25], *meso*-dihydroguaiaretic acid (**2**) [26,27], and machilin A (**3**) [28,29], by ^1^H-NMR, ^13^C-NMR, and MS spectral data and compared them with previously reported physical and spectroscopic data (Figure 1).

In order to evaluate the antiallergic activity of compounds **1**–**3** of the bark of *M. thunbergii*, an assay was performed to confirm the inhibitory effect of STAT5 phosphorylation and binding between TSLP and TSLPR through pSTAT5 assay and ELISA assay, respectively. TSLP, a cytokine involved in the initiation of allergic reactions, has been reported as the only cytokine that characteristically phosphorylates STAT5 among STAT proteins using a combination of JAK1 and JAK2 linked to the IL-7Rα and TSLPR [30].

Compounds **1** and **2** showed strong pSTAT5 inhibitory activities of 54.5% and 64.1% at 30 µM, respectively, in HMC-1 cells stimulated with hTSLP (Table 1 and Figure S1). None of them have reported pSTAT5 inhibitory activity. Song et al. investigated the antiasthmatic activity of *meso*-dihydroguaiaretic acid (**2**) in an ovalbumin (OVA)-induced allergic asthma model and found that it decreased the levels of Th2-type cytokines and total immunoglobulin (Ig) E [31]; however, the antiallergic activity of (+)-galbelgin (**1**) was first identified in our study. In addition, as shown in Table 2, compound **1** showed more than 20% inhibitory activity of hTSLP–hTSLPR interaction at 0.3 mM, which correlated with the pSTAT5 assay. However, the other two compounds (**2** and **3**) did not show more than 20% inhibitory activity at 0.3 mM. These results suggested compound **1** as a material that can fundamentally prevent and treat allergic diseases by inhibiting TSLP-mediated signal transduction.

**Figure 2:**
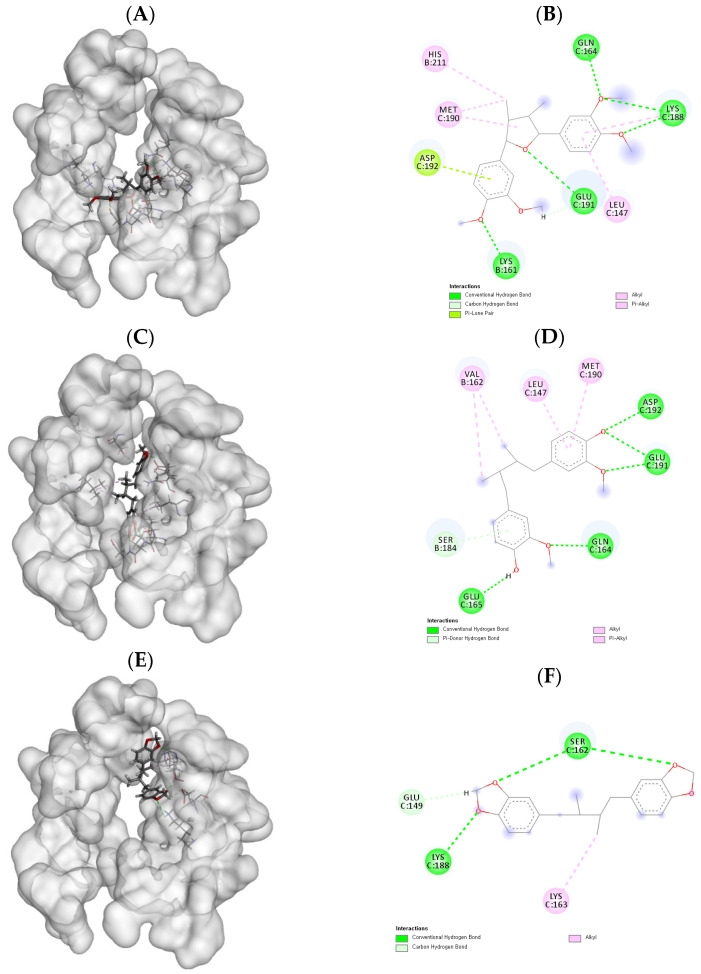
Molecular docking results of isolated compounds **1**–**3** against human TSLP in complex with TSLPR and interleukin-7 receptor α chain (IL-7Rα): (**A**) three-dimensional (3D) docking image of compound **1**; (**B**) two-dimensional (2D) docking image of compound **1**; (**C**) 3D docking image of compound **2**; (**D**) 2D image picture of compound **2**; (**E**) 3D docking image of compound **3**; (**F**) 2D docking picture of compound **3**.

To further elucidate the inhibitory activity of the isolated compounds against hTSLP–hTSLPR interaction, molecular docking studies were conducted for human TSLP in complex with TSLPR and IL-7Rα. As shown in Table 3, compound **1** had the highest docking score compared to the other compounds, which is consistent with the ELISA results. Moreover, as shown in Figure 2A, compound **1** appeared to be bound to the hTSLP–hTSLPR interaction interface. The interaction mode of compound **1** is shown in Figure 2B. Six hydrogen bonds were formed between the 3′, 4, 3, and 4″-methoxy oxygen and 1-oxygen of compound **1** with Lys161, Lys188, Gln164, and Glu191, respectively. The lengths of these hydrogen bonds were 1.89, 1.88, 2.46, 2.13, 2.13, and 2.43 Å. As shown in Figure 2C,D, six hydrogen bonds were formed between 3, 3′-methoxyl oxygen and 4, 4′-OH and Gln164, Glu165, Asp192, Glu191, and Ser184, with lengths of 1.91, 1.93, 2.07, 2.09, 3.05, and 2.95 Å, respectively. On the other hand, compound **3** formed four hydrogen bonds between methylenedioxy oxygen atoms and Ser162, Glu149, and Lys188, with lengths of 1.93, 2.08, 2.56, and 2.95 Å, respectively (Figure 2E,F). These results demonstrate that these compounds are able to bind tightly to catalytic amino acid residues to inhibit hTSLP–hTSLPR interaction. Furthermore, compounds with more hydroxyl and methoxy groups exhibited more hydrogen bonding and higher docking scores. As a high-scoring inhibitor of the hTSLP–hTSLPR interaction, compound **1** is a good candidate for the treatment of allergic diseases, consistent with the ELISA results.

## 3. Materials and Methods

### 3.1. General Experimental Procedures

Intracellular phospho-STAT5 (pSTAT5) was measured using a BD LSR Fortessa Flow cytometer (BD Biosciences, San Diego, CA, USA). NMR spectra were recorded on a Bruker SPECTROSPIN 300 MHz spectrometer (Bruker Corporation, Billerica, MA, USA). For the pSTAT5 assay measuring TSLP inhibitory activity, recombinant human TSLP was purchased from R&D Systems (Minneapolis, MN, USA) and BD Cytofix/Cytoperm and Alexa Fluor 647 mouse anti-STAT5 (pY694) were purchased from BD Biosciences (San Diego, CA, USA). For the ELISA assay to assess hTSLP–hTSLPR interaction, Ni-NTA HisSorb plates were purchased from Qiagen (Hilden, Germany), and monoclonal anti-FLAG antibody conjugated to HRP and *o*-phenylenediamine dihydrochloride were purchased from Sigma-Aldrich Co. (St. Louis, MO, USA). Iscove’s modified Dulbecco’s medium (IMDM) and fetal bovine serum (FBS) were purchased from Hyclone Laboratories Inc. (Logan, UT, USA). Penicillin-streptomycin was purchased from Gibco Industries Inc. (Auckland, NZ, USA).

### 3.2. Plant Material

The bark of *M. thunbergii* was obtained from the Medicinal Herb Garden, College of Pharmacy, Seoul National University, Seoul, Korea, and identified by Dr. Ki Yong Lee, a professor at the College of Pharmacy, Korea University (Sejong, Korea). The voucher specimen (KUP-HD066) was deposited at the Laboratory of Pharmacognosy, College of Pharmacy, Korea University.

### 3.3. Extraction and Isolation

The bark of *M. thunbergii* (500 g) was extracted three times with 80% MeOH under ultrasonic conditions for 90 min. After removing the solvent in vacuo, the obtained crude extracts (45.2 g) were suspended in distilled water and partitioned using dichloromethane to yield a dichloromethane fraction (7.1 g). The dichloromethane fraction was subjected to medium-pressure liquid chromatography (MPLC) on a silica gel column using an Isolera One system (Biotage, Uppsala, Sweden) and eluted with an *n*-hexane:EtOAc:MeOH gradient (20:1:0→0:0:1) to yield 32 fractions (D1–D32). Compound **1** (2.3 mg) was purified from D20 via recrystallization. Fractions D5 and D7 were separated using Sephadex LH-20 column chromatography. Compounds **2** (12.3 mg) and **3** (2.1 mg) were obtained from D7-2 and D5-4, respectively, and were observed as a single spot on a TLC plate.

### 3.4. pSATA5 Assay

#### 3.4.1. Cell Culture

HMC-1 cells were obtained from the Department of Food Technology and Inflammatory Disease Research Center, Hoseo University, Asan, Chungnam, Korea. HMC-1 cells were cultured in IMDM containing 10% FBS and 1% penicillin-streptomycin at 37 °C under a humidified atmosphere of 5% CO_2_ and 95% air. Subculturing was performed every 2 days. When HMC-1 cells were grown to 80% confluence, the cells were harvested and centrifuged at 1000 rpm for 3 min. Then, the cells were recultured with a complete growth medium in a 1:3 volume ratio.

#### 3.4.2. Flow Cytometry

pSTAT5 staining was performed according to the protocol obtained from the laboratory of Susan Kaech, Department of Immunobiology, Yale University School of Medicine, New Haven, CT, USA. Briefly, HMC-1 cells were seeded at a density of 1 × 10^7^ cells/mL in a 96-well U-bottom plate, and the supernatant was discarded after centrifugation (1650 rpm for 2 min). The cells were then stimulated with 100 ng/mL of recombinant hTSLP alone (control) or with the sample for 30 min and fixed in the BD Cytofix/Cytoperm solution for 10 min. The fixed cells were permeabilized in ice-cold methanol for 30 min. After permeabilization, intracellular pSTAT5 was stained with Alexa Fluor 647 mouse anti-STAT5 for 30 min. The stained cells were washed twice in FACS buffer and resuspended in the FACS buffer before flow cytometry analysis. pSTAT5 was acquired on a BD LSRFortessa flow cytometer and analyzed using FlowJo software (version 9.7.6) (Tree Star Inc., Ashland, OR, USA).

### 3.5. ELISA Assay

ELISA was performed according to a previously described method [4]. The C-terminal octa-histidine tag (TSLPR-His) and N-terminal FLAG tag (FLAG-hTSLP) were obtained from Dr. Young Ho Jeon, a professor at the College of Pharmacy, Korea University, Sejong, Korea. A solution containing hTSLPR with C-terminal octa-histidine tag (TSLPR-His) was added to each well at a rate of 100 µL. After incubation at a room temperature for 2 h, the plate was washed three times with 200 µL of PBS containing 0.05% Tween-20 to remove unbound TSPL-His. Potential inhibitors were treated with concentrations of 0.3 and 0.1 mM, and 100 µL of TSLP with N-terminal FLAG tag (FLAG-hTSLP) was added and incubated overnight (18 h) at 4 °C. The plate was washed three times to remove unbound FLAG-hTSLP. Next, the plate was blocked with the 100 µL blocking buffer (PBS with 0.05% Tween-20, 1% non-fat dry milk, and 1% bovine serum albumin) for 2 h and washed three times. Then, the plate was coated with 100 µL of monoclonal anti-FLAG antibody conjugated to HRP and incubated for 2 h. The incubated plate was washed five times, and 200 µL of an *o*-phenylenediamine dihydrochloride solution were added and incubated for 30 min. To stop the reaction, 1N HCl was added to the plate, and the inhibitory effect was determined by measuring the optical density (OD) at 450 nm in a 96-well microplate reader. The inhibitory effects were calculated using the following equation:

Inhibitory effect (%) = (1 − OD of sample/OD of control) × 100%.(1)

### 3.6. Molecular Docking Studies

Molecular docking studies were conducted using SYBYL-X (version 2.1.1) (Tripos Ltd., St. Louis, MO, USA). The structure of human TSLP in complex with TSLPR and IL-7Ralpha (PDB-ID: 5J11) was provided by the Research Collaboratory for Structural Bioinformatics Protein Data Bank (RCSB PDB). The protein was prepared with a Tripos force field and a termination of 0.05, and all water compounds were removed. The three-dimensional (3D) structures of the ligands were prepared using Chem3D Pro and saved as mol files. Molecular docking was performed using the Surflex preparation protocol in SYBYL-X 2.1.1, and the docking results were analyzed by Discovery Studio 2019 Client (Biovia Co., San Diego, CA, USA), which showed the interaction between ligands and residues in a docked complex.

## 4. Conclusions

The antiallergic activity of lignans isolated from the bark of *M. thunbergii* was evaluated. Among these, (+)-galbelgin (**1**) and *meso*-dihydroguaiaretic acid (**2**) were found to exhibit strong pSTAT5 and TSLP/TSLPR interaction inhibitory activity in an in vitro assay (STAT5 assay and ELISA assay) and an in silico assay (docking study). Notably, (+)-galbelgin (**1**), the most bioactive compound, was identified as an antiallergic agent for the first time in this study. Therefore, lignans of the bark of *M. thunbergii* may be a good resource for the development of antiallergic agents targeting the TSLP signaling pathway.

## Figures and Tables

**Table 1:** The effect of isolated compounds on STAT5 phosphorylation by flow cytometry.

	STAT5 Phosphorylation (%)	
	3 μM	30 μM
**Extracts of *M. thunbergii***	79.2 ^(a)^	69.2 ^(b)^
**1**	67.6	54.5
**2**	68.7	64.1
**3**	111.9	104.3

^(a)^ 50 µg/mL; ^(b)^ 100 µg/mL.

**Table 2:** The inhibitory effects of isolated compounds **1**–**3** on thymic stromal lymphopoietin (TSLP)/TSLP receptor (TSLPR) interaction by ELISA.

Compounds	hTSLP/hTSLPR Interaction Inhibition (%) ^a^	
	0.1 mM	0.3 mM
Control	0.0 ± 0.9	
**1**	17.2 ± 2.2 **	27.3 ± 1.9 *
**2**	13.8 ± 0.6 **	16.7 ± 1.2 **
**3**	11.0 ± 1.5 **	19.5 ± 1.2 *

^a^ Samples that differ significantly from the control. * *p* < 0.05, ** *p* < 0.01.

**Table 3:** Molecular docking results of isolated compounds **1**–**3** on TSLP/TSLPR interaction.

Compounds	Docking Analysis (Total Score) ^a^	Key Residues
**1**	7.4243	Lys161(1.89), Lys188(1.88, 2.46), Gln164(2.13), and Glu191(2.13, 2.43)
**2**	7.2410	Gln164(1.91), Glu165(1.93), Asp192(2.07), Glu191(2.09, 3.05), and Ser184(2.95)
**3**	5.7852	Ser162(1.93, 2.08), Glu149(2.56), and Lys188(2.95)

^a^ Total scores were expressed in –log10(*K_d_*)^2^ units to represent binding affinities.

## Supplementary Materials

The following are available, Figure S1: Flow cytometric analysis of STAT5 phosphorylation levels in hTSLP-stimulated HMC-1 cells.

## Sample Availability

Samples of the compounds are not available from the authors.

## References

[1] Adamu R.M., Malik B.K. (2018). Molecular Modeling and Docking Assessment of Thymic Stromal Lymphopoietin for the Development of Natural Anti Allergic Drugs. J. Young. Pharm..

[2] Han N.R., Moon P.D., Kim H.M., Jeong H.J. (2014). Tryptanthrin ameliorates atopic dermatitis through down-regulation of TSLP. Arch. Biochem. Biophys..

[3] Moon P.D., Jeong H.J., Kim H.M. (2012). Effects of schizandrin on the expression of thymic stromal lymphopoietin in human mast cell line HMC-1. Life Sci..

[4] Park B.B., Choi J.W., Park D., Choi D., Paek J., Kim H.J., Son S.Y., Mushtaq A.U., Shin H., Kim S.H. (2019). Structure-activity relationships of baicalein and its analogs as novel TSLP inhibitors. Sci. Rep..

[5] Zhong J., Sharma J., Raju R., Palapetta S.M., Prasad T.S., Huang T.C., Yoda A., Tyner J.W., van Bodegom D., Weinstock D.M. (2014). TSLP signaling pathway map: A platform for analysis of TSLP-mediated signaling. Database.

[6] Bantz S.K., Zhu Z., Zheng T. (2014). The Atopic March: Progression from Atopic Dermatitis to Allergic Rhinitis and Asthma. J. Clin. Cell Immunol..

[7] Spergel J.M. (2010). From atopic dermatitis to asthma:the atopic march. Ann. Allergy Asthma Immunol..

[8] Kapoor Y., Kumar K. (2020). Structural and clinical impact of anti-allergy agents: An overview. Bioorg. Chem..

[9] Smruti P. (2021). A review on natural remedies used for the treatment of respiratory disorders. Int. J. Pharm..

[10] Wu S.-J., Len W.-B., Huang C.-Y., Liou C.-J., Huang W.-C., Lin C.-F. (2015). *Machilus thunbergii* extract inhibits inflammatory response in lipopolysaccharide-induced RAW264.7 murine macrophages via suppression of NF-κB and p38 MAPK activation. Turk. J. Biol..

[11] Ren Q., Wu D., Wu C., Wang Z., Jiao J., Jiang B., Zhu J., Huang Y., Li T., Yuan W. (2020). Modeling the Potential Distribution of *Machilus thunbergii* under the Climate Change Patterns in China. Open J. For..

[12] Ma C.J., Kim Y.C., Sung S.H. (2009). Compounds with neuroprotective activity from the medicinal plant *Machilus thunbergii*. J. Enzym. Inhib. Med. Chem..

[13] Jeon J.S., Oh S.J., Lee J.Y., Ryu C.S., Kim Y.M., Lee B.H., Kim S.K. (2015). Metabolic characterization of meso-dihydroguaiaretic acid in liver microsomes and in mice. Food Chem. Toxicol..

[14] Kim W., Lyu H.N., Kwon H.S., Kim Y.S., Lee K.H., Kim D.Y., Chakraborty G., Choi K.Y., Yoon H.S., Kim K.T. (2013). Obtusilactone B from *Machilus thunbergii* targets barrier-to-autointegration factor to treat cancer. Mol. Pharm..

[15] Lee M.K., Yang H., Ma C.J., Kim Y.C. (2007). Stimulatory activity of lignans from *Machilus thunbergii* on osteoblast differentiation. Biol. Pharm. Bull..

[16] Ma C.J., Kim S.R., Kim J., Kim Y.C. (2005). Meso-dihydroguaiaretic acid and licarin A of *Machilus thunbergii* protect against glutamate-induced toxicity in primary cultures of a rat cortical cells. Br. J. Pharm..

[17] Karikome H., Mimaki Y., Sashida Y. (1991). A butanolide and phenolics from *Machilus thunbergii*. Phytochemistry.

[18] Yu Y.U., Kang S.Y., Park H.Y., Sung S.H., Lee E.J., Kim S.Y., Kim Y.C. (2000). Antioxidant Lignans from *Machilus thunbergii* protect CCl4-injured primary cultures of rat hepatocytes. J. Pharm. Pharm..

[19] Ma C.J., Lee M.K., Kim Y.C. (2006). Meso-dihydroguaiaretic acid attenuates the neurotoxic effect of staurosporine in primary rat cortical cultures. Neuropharmacology.

[20] Ma C.J., Sung S.H., Kim Y.C. (2010). New neuroprotective dibenzyl butane lignans isolated from *Machilus thunbergii*. Nat. Prod. Res..

[21] Favela-Hernandez J.M., Garcia A., Garza-Gonzalez E., Rivas-Galindo V.M., Camacho-Corona M.R. (2012). Antibacterial and antimycobacterial lignans and flavonoids from *Larrea tridentata*. Phytother. Res..

[22] Le T.V.T., Nguyen P.H., Choi H.S., Yang J.-L., Kang K.W., Ahn S.-G., Oh W.K. (2017). Diarylbutane-type lignans from *Myristica fragrans* (Nutmeg) show the cytotoxicity against breast cancer cells through activation of AMP-activated protein kinase. Nat. Prod. Sci..

[23] Seo S.G., Park D.J., Cho H.S., Kim G.N., Kim J.H., Park J.H., Jin M.H. (2021). Compositions Containing Paper Mulberry Extracts. Patent.

[24] Liu J.-S., Huang M.-F., Gao Y.-L. (1981). The structure of chicanine, a new lignan from *Schisandra* sp.. Can. J. Chem..

[25] Rye C.E., Barker D. (2011). Asymmetric synthesis of (+)-galbelgin, (-)-kadangustin J, (-)-cyclogalgravin and (-)-pycnanthulignenes A and B, three structurally distinct lignan classes, using a common chiral precursor. J. Org. Chem..

[26] Nakatani N., Ikeda K., Kikuzaki H., Kido M., Yamaguchi Y. (1988). Diaryldimethylbutane Lignans from Myristica argentea and their antimicrobial action against *Streptococcus mutans*. Phytochemistry.

[27] Hwu J.R., Tseng W.N., Gnabre J., Giza P., Huang R.C.C. (1998). Antiviral activities of methylated nordihydroguaiaretic acids. 1. Synthesis, structure identification, and inhibition of Tat-regulated HIV transactivation. J. Med. Chem..

[28] Shimomura H., Sashida Y., Oohara M. (1987). Lignans from *Machilus thunbergii*. Phytochemistry.

[29] Lee S.U., Shim K.S., Ryu S.Y., Min Y.K., Kim S.H. (2009). Machilin A isolated from *Myristica fragrans* stimulates osteoblast differentiation. Planta Med..

[30] Rochman Y., Kashyap M., Robinson G.W., Sakamoto K., Gomez-Rodriguez J., Wagner K.U., Leonard W.J. (2010). Thymic stromal lymphopoietin-mediated STAT5 phosphorylation via kinases JAK1 and JAK2 reveals a key difference from IL-7–induced signaling. Proc. Natl. Acad. Sci. USA.

[31] Song J.W., Seo C.S., Cho E.S., Kim T.I., Won Y.S., Kwon H.J., Son J.K., Son H.Y. (2016). Meso-dihydroguaiaretic acid attenuates airway inflammation and mucus hypersecretion in an ovalbumin-induced murine model of asthma. Int. Immunopharmacol..

