# Supplementary material for: Lignans from Machilus thunbergii as Thymic Stromal Lymphopoietin Inhibitors
Source: Molecules. 2021 Aug 8;26(16):4804. doi: 10.3390/molecules26164804 (PMC8398558; doi:10.3390/molecules26164804)
Supplement: Supplementary file 1 [file molecules-26-04804-s001.zip › molecules-1307523-supplementary.pdf]

Supporting information

Lignans from *Machilus thunbergii* as thymic stromal lymphopoietin inhibitors

Hyeji Shin[1,#], Yoo Kyong Han[1,#], Youngjoo Byun[1], Young Ho Jeon[1], and Ki Yong Lee[1,*]

[1] College of Pharmacy, Korea University, Sejong, Republic of Korea; hjshin90@korea.ac.kr (H.S); kkoo_@naver.com (Y.K.H); yjbyun1@korea.ac.kr (Y.B); yhjeon@korea.ac.kr (Y.H.J)

[#] These authors contributed equally to this work

[*] Correspondence: kylee11@korea.ac.kr (K.Y.L.); Tel.: +82-44-860-1623 (K.Y.L)

(A)

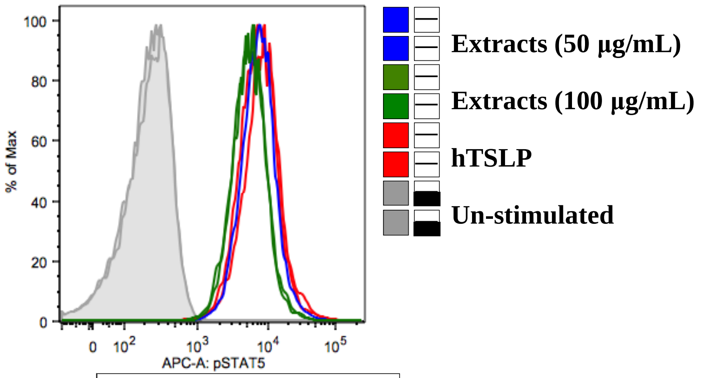

(B)

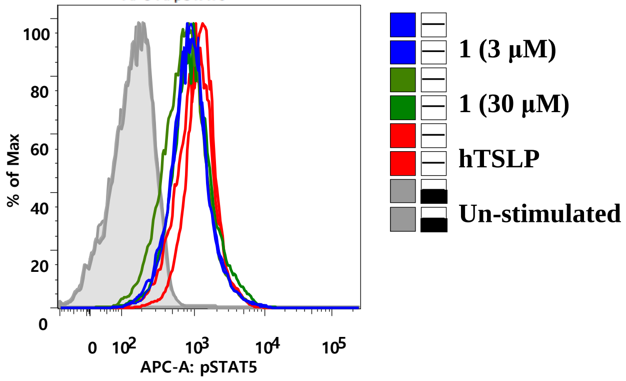

(C)

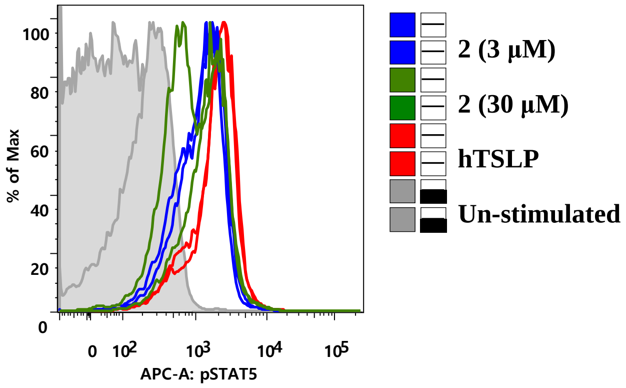

(D)

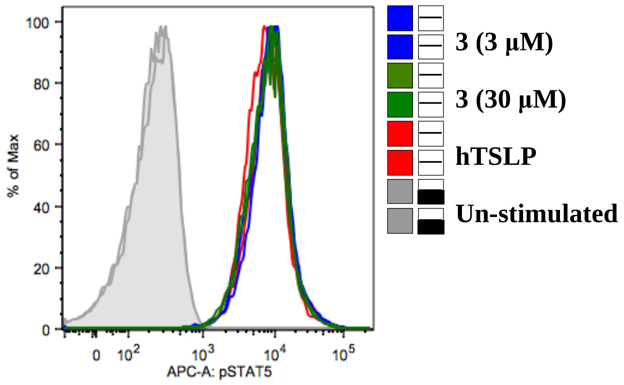

Figure S1. Representative flow cytometry histogram of STAT5 phosphorylation. (**A**) Extracts of *M. thunbergii*; (**B**) compound **1**; (**C**) compound **2**; (**D**) compound **3** .
